# Exercise as a protective mechanism against the negative effects of oxidative stress in first-episode psychosis: a biomarker-led study

**DOI:** 10.1038/s41398-020-00927-x

**Published:** 2020-07-24

**Authors:** Emily Fisher, Stephen J. Wood, Richard J. Elsworthy, Rachel Upthegrove, Sarah Aldred

**Affiliations:** 1 School of Sport, Exercise and Rehabilitation Sciences, University of Birmingham, Edgbaston, B15 2TT UK; 2 Orygen, The National Centre of Excellence in Youth Mental Health, Parkville, Melbourne, VIC 3052 Australia; 3 School of Psychology, University of Melbourne, Melbourne, VIC 3010 Australia; 4 Centre for Youth Mental Health, University of Melbourne, Melbourne, VIC 3010 Australia; 5 Institute for Mental Health, University of Birmingham, Edgbaston, B15 2TT UK; 6 Department of Psychiatry, University of Birmingham, Edgbaston, B15 2TT UK

**Keywords:** Physiology, Human behaviour, Molecular neuroscience, Diagnostic markers

## Abstract

First-episode psychosis (FEP) is a psychiatric disorder, characterised by positive and negative symptoms, usually emerging during adolescence and early adulthood. FEP represents an early intervention opportunity for intervention in psychosis. Redox disturbance and subsequent oxidative stress have been linked to the pathophysiology of FEP. Exercise training can perturb oxidative stress and rebalance the antioxidant system and thus represents an intervention with the potential to interact with a mechanism of disease. The aim of this study was to assess the effect of exercise on markers of redox status in FEP. Twenty-two young men were recruited from Birmingham Early Intervention services and randomised to either a 12-week exercise programme or treatment as usual (control). Measures of blood and brain glutathione (GSH), markers of oxidative damage, inflammation, neuronal health, symptomology and habitual physical activity were assessed. Exercise training was protective against changes related to continued psychosis. Symptomatically, those in the exercise group showed reductions in positive and general psychopathology, and stable negative symptoms (compared to increased negative symptoms in the control group). Peripheral GSH was increased by 5.6% in the exercise group, compared to a significant decrease (24.4%) (*p* = 0.04) in the control group. Exercise attenuated negative changes in markers of neuronal function (brain-derived neurotrophic factor), lipid damage (thiobarbituric acid-reactive substances) and total antioxidant capacity. C-reactive protein and tumour necrosis factor-α also decreased in the exercise group, although protein and DNA oxidation were unchanged. Moderate-intensity exercise training has the ability to elicit changes in markers of oxidative stress and antioxidant concentration, with subsequent improvements in symptoms of psychosis.

## Introduction

First-episode psychosis (FEP) is a psychiatric disorder, characterised by positive and negative symptoms, usually emerging during adolescence and early adulthood^1^. It is well described that a delay in FEP treatment can lead to cognitive alterations and worsened functional outcome^2^. FEP patients are highly heterogeneous in symptom profile, treatment response and cannabis consumption, and there is a need for improved characterisation of biomarkers related to disease pathophysiology in order to better understand pathology of disease. Many of the biochemical perturbations observed in this population are associated with the psychosis phenotype and thus represent targets for treatment.

Several aspects of (neuro) physiology are altered in FEP and schizophrenia patients, including dysfunctional neurotransmitter systems^3,4^, decreased synaptic plasticity^5^ and reduced hippocampal volume^6^. In addition, inflammation and cellular redox status is also altered. This is often termed oxidative stress, and represents an imbalance between reactive radicals or oxidants, which are naturally produced in aerobic metabolism, and antioxidants, which balance and quench oxidants to allow essential signalling processes, but prevent damage to biomolecules leading to cell injury or death^7^. Brain-derived neurotrophic factor (BDNF), which has an essential role in neural signal transmission and synaptic plasticity, is depleted in FEP^8^. It has also been suggested that BDNF is responsible for protecting against oxidative stress by upregulating the expression of antioxidant enzymes^9^, highlighting the value of BDNF as a candidate biomarker of psychosis.

Glutathione (GSH) is the most abundant antioxidant that acts as a scavenger of reactive oxygen species, with a primary role of maintaining the intracellular redox balance^10^. GSH has been implicated in the pathogenesis of a range of neurodegenerative and psychotic diseases^11^. FEP patients have shown as much as a 52% reduction in GSH compared with controls^12,13^. Indeed, evidence of perturbed redox homeostasis in FEP and schizophrenia is plentiful. There have been reports of reduced total GSH^13–15^, increased oxidised GSH (GSSG)^15^, increased lipid^16,17^, protein^18,19^ and DNA damage^20,21^. However, conflicting data by Reddy et al.^22^ also showed no impairment of antioxidant defences in comparison with a healthy control group, hypothesising that disease stage is related to progression of oxidative stress accumulation. A study by Fraguas et al.^14^ assessed the relationship between grey matter volume and GSH in the brains of schizophrenia patients. The study highlighted a progressive blood GSH decline over the 2-year follow-up period, as well as a relationship between grey matter loss and blood GSH.

The brain is a particularly vulnerable target for free-radical-mediated damage due to a high level of oxygen consumption^23,24^, and high lipid content^19^. When this high oxygen concentration is coupled with a modest endogenous antioxidant concentration^25^, and inability of GSH to cross the blood–brain barrier (BBB), particularly from exogenous supplementation or circulating pools, the antioxidant defence system is stretched. Assessment of brain redox state in vivo is not easy, and peripheral markers are usually employed as an estimate of whole-body oxidative environment and antioxidant concentration. However, recent methodological advances in magnetic resonance spectroscopy (MRS) have allowed the quantification of GSH in the cerebral tissue^26^.

Exercise has been identified as an adjunctive treatment for psychosis as it has the ability to improve clinical symptoms^27^ and has the potential to restore redox homeostasis, as demonstrated in healthy populations^28^. However, no study has proven the efficacy of exercise in altering the underlying pathology of disease. Studies employing moderate-intensity aerobic exercise^29^ and combined aerobic and resistance training^30^ across 16 and 12 weeks, respectively, have resulted in reduced psychotic symptoms assessed by the Positive and Negative Symptom Scale (PANSS). Acil et al.^31^ described a 14% increase in quality of life, as well as a decrease of positive and negative symptoms that define the schizophrenic phenotype.

Mechanisms underpinning exercise adaptation are well established in studies of healthy participants and are characterised by an antioxidant response to radical signals^32^. Elokda and Nielsen^33^ demonstrated adaptations to either aerobic training or combined aerobic and circuit weight training (standardised workload). Both training programmes led to a significant increase in GSH, and reduced oxidised GSH (GSSG). Additionally, exercise training has the potential to modify other indices of redox status. Studies in health volunteers have shown that exercise training may elevate BDNF^34^ and reduce oxidative damage markers, including malondialdehyde (lipid peroxidation) and protein carbonyl concentration^35^.

Furthermore, exercise training is beneficial in reducing circulating proinflammatory cytokines^36^. There is robust evidence to implicate a systemic proinflammatory state in FEP. Inflammation and oxidative stress are strongly linked in a number of pathologies. Systematic review^37^ confirms elevated proinflammatory cytokines in drug-naive FEP, including interleukin-6 (IL-6), tumour necrosis factor-alpha (TNF-α), IL-1β and sIL-2R, and there is clear evidence of elevated IL-6 in childhood, predicting risk for both psychosis and metabolic dysfunction. Regular physical activity can lead to reductions in circulating IL-6^38,39^, TNF-α^40^ and C-reactive protein (CRP)^41,42^.

The aim of this exploratory pilot study was to assess the effect of 12 weeks of exercise training on the GSH system in FEP, via in vivo brain and peripheral blood measures. To add context around changes in GSH, measures of oxidative damage, inflammation and neuronal health were assessed, as well as symptoms of psychosis.

## Methods

### Participants

Male patients, aged 16–35 years, with a diagnosis of FEP (as identified by a psychiatrist in keeping with ICD-10 F 20-29, F31.2, 32.3 diagnostic criteria), were recruited from the community-based Birmingham Early Intervention service. Patients were within 3 years of first presentation of illness. Male patients only were recruited, since the oestrogen cycle has a significant effect on antioxidant concentration, in particular GSH^43^. Eligibility criteria were assessed initially by the primary care coordinators for each patient, followed by assessment of habitual activity to ensure a sedentary lifestyle, as well as the use of a general health questionnaire to assess cannabis use and confirm the patient was free from medical conditions that prevent participation in moderate-intensity aerobic exercise. A sample size of 28 was calculated, using the G*Power software^44^, based on intervention-associated change in GSH. Exclusion criteria included failure to adhere to pretesting requirements, for example, provide a blood sample, or significant risk to self of others as identified by the clinical team. Study assessments took place at baseline, mid-point (for the exercise group only) and post intervention. This study was commenced following approval from the NIHR HRA ethics committee (West Midlands- Edgbaston REC 17/WM/0412). Intervention design, quality and patient-oriented outcomes were assessed and are summarised by Fisher et al.^45^.

### Randomisation

Following consent, participants were randomised to either the exercise intervention group or the control arm (treatment as usual) of the study. A block randomisation method (http://www.randomization.com)^46,47^ was used to allow for equal group distribution in the event of poor recruitment.

### Exercise intervention

Exercise sessions were designed and supervised by a trained researcher at the School of Sport, Exercise and Rehabilitation Sciences, University of Birmingham. The intervention was 12 weeks long, with each participant required to exercise at least 2 times per week, for 40–60 min per session. In an effort to maximise attendance and compliance, which has historically been difficult in this group^48^, participants were given a choice of different activities to undertake at each session (available exercise: running; cycling; swimming; tennis; squash; badminton; circuit training and football). Each training session was standardised by heart rate target zone, based on 70–80% HRmax (maximum heart rate). The minimum training intensity for improvement in aerobic fitness is 55–65% HRmax^49^; therefore, in order to observe a meaningful and significant effect of exercise, intensity was set above this. The ACSM (American College of Sports Medicine) recommends 50% *V*O_2_max (maximum rate of oxygen consumption) as a minimum intensity for exercise training^50^, which corresponds to 65% HRmax. The relationship between HR and VO_2_ reflect energy expenditure in a linear fashion, up to 85% HRmax^51^. Energy consumption, resting heart rate, active calories and intensity minutes (equivalent to moderate-intensity exercise) was tracked at three time-points in the intervention period (baseline, mid-intervention and post intervention), and for the duration of each exercise bout by Garmin VivoSmart™ HR activity monitor (Garmin, USA).

### Blood sampling

Cephalic/cubital venous blood samples were taken at pre-intervention (0 weeks), mid-intervention (6 weeks) and post-intervention (13 weeks) time-points, into BD Vacutainer® MAP K_2_EDTA 1.0 mg tubes (BD, USA). Following removal of whole blood (2 × 1 mL) for comet assay and GSH analysis, samples were centrifuged (2000 r.p.m., 15 min, 10 °C) and the resultant plasma was aliquoted for subsequent determination of FRAP (ferric-reducing ability of plasma), TBARS (thiobarbituric acid-reactive substances), 8-isoprostane, BDNF, IL-6, CRP, TNF-α and protein carbonyl. All samples were stored at −80 °C for a maximum of 9 months until analysis.

### MRS GSH measurement

**Fig. 1:**
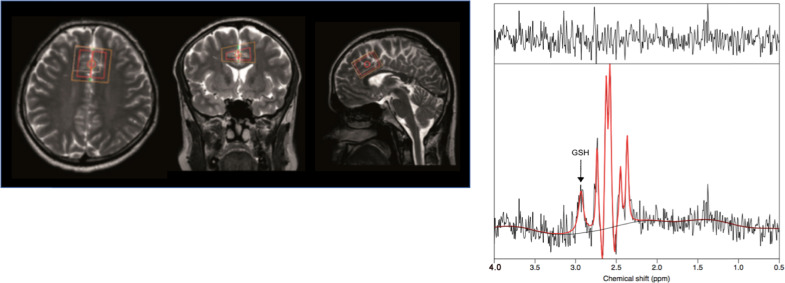
T1 weighted MRI image demonstrating the volume of interest (VOI) pictured. 30 × 30 × 20 mm. Placed in the anterior cingulate cortex. Image above represents axial, frontal and sagittal views from left to right. The metabolite peak on the right shows an example, edited spectra with labelled glutathione peak at 2.95 mm.

Scans were conducted at the Birmingham University Imaging Centre, using a 3 Tesla Phillips Achieva MRI (magnetic resonance imaging) scanner, with a 32-channel head coil. The ^1^H single-voxel Mescher–Garwood point-resolved spectroscopy method of acquisition was employed, with repetition time = 2 s; echo time = 131 ms; 55 Hz bandwidth editing pulse at 4.56 p.p.m.; and 1024 complex data points acquired at a sampling frequency of 2000 Hz, followed by water suppression. The MRS protocol included a T1-weighted structural MRI for MRS planning (5 min). The volume of interest was located in the anterior cingulate cortex (30 × 30 × 20 mm^3^) (Fig. 1). Total MRS scan time for GSH measurement was 18 min. Spectral alignment was completed using the RATS (Robust Alignment to a Target Spectrum) method^52^, implemented in R (v3.5.0) (Vienna, Austria), and integrated into the SPANT (SPectroscopy ANalysis Tools) package (v0.12.0) for MRS analysis. GSH was then fitted using TARQUIN. An example spectrum is detailed in Fig. 1, following post-processing, with the GSH peak highlighted.

### Blood GSH

Whole-blood GSH concentration was determined using a commercially available luminescence-based assay (GSH-Glo™ Glutathione Assay, Promega, WI, USA). The assay was undertaken according to the manufacturer’s instructions.

### Comet assay

Cells were prepared for assessment of single-strand DNA strand breaks, characterised by Singh et al.^53^. Thawed whole-blood samples were used for analysis, breaking away from the conventional use of isolated mononuclear cells, replicating the protocol described by Akor-Dewu et al.^54^. Active SYBR-GOLD nucleic acid gel staining solution (1:1000 dilution of stock solution in neutralisation buffer) was distributed across the slides and incubated at room temperature before scoring (tail length representing single-strand break density), using a fluorescent microscope (Zeiss Axiovert 10, Germany).

### Lipid peroxidation

8-Isoprostane and TBARS concentrations were used to assess lipid peroxidation in the plasma. For TBARS, plasma samples and standards (1,1,3,3-tetramethoxypropane) (100 µl) were mixed with trichloroacetic acid (410 mM, 100 µl) and colour reagent (4.6 mM thiobarbituric acid, 1.74 M glacial acetic acid and 0.67 M butylated hydroxytoluene, 800 µl) in Eppendorf tubes. After boiling vigorously in water (100 °C) for 1 h, the reaction was stopped by placing the tubes in an ice bath for 10 min. Supernatants were transferred to a multiwell plate and the absorbance was measured at 540 nm. The concentration of 8-isoprostane in plasma samples was determined by competitive enzyme-linked immunosorbent assay using a commercially available kit (8-isoprostane EIA Kit 516351, Cayman Chemical, Ann Arbor, MI), according to the manufacturer’s instructions. After pretreatment of the plasma sample to concentrate the total 8-isoprostane content, affinity sorbent (8-isoprostane affinity sorbent 401113-1, Cayman Chemical, Ann Arbor, MI) was used.

### Protein carbonyls

Protein carbonyl concentration was determined in the plasma. Anti-DNP (2,4-dinitrophenol) antiserum primary antibody (1:1000) and peroxidase-labelled secondary antibody (rat anti-mouse IgE in blocking buffer 1:5000) were used, following the protocols described by Buss et al.^55^ and Alamdari et al.^54^.

### Inflammatory markers

Plasma IL-6, CRP and TNF-α concentrations were determined using commercially available immunoassay kits (Human IL-6 Quantikine ELISA Kit (D6050), Human TNF-alpha Quantikine ELISA Kit (DTA00C) and Human C-Reactive protein/CRP Quantikine ELISA Kit (DCRP00)), from R&D Systems (Minnesota, USA). Concentrations were determined according to the manufacturer’s instructions.

### Total antioxidant capacity using the FRAP method

The FRAP method was developed by Benzie and Strain^56^. Standards were prepared using a 0–1000 μM concentration range of ascorbic acid. FRAP reagent—consisting of acetate buffer (300 mM sodium acetate at pH 3.6 (3.1 g) into neat 16 mL glacial acid per litre of buffer solution), TPTZ (2, 4, 6-Tris (2-pyridyl)-*S*-triazine) solution (160 mM: 0.05 g/mL–0.1 g TPTZ in 2 mL methanol, then 2 mL into 30 mL 40 mM HCl) and FeCl_3_·6H_2_O solution (0.332 g ferric chloride in 100 mL ddH_2_O)—was added to samples/standards on the plate, incubated for 8 min at room temperature and read at 650 nm. FRAP concentrations were determined by linear regression relative to ascorbic acid.

### Brain-derived neurotrophic factor

Plasma BDNF concentration was determined using a commercially available immunoassay kit (Human BDNF ELISA Kit (ab99978), Abcam, Cambridge, UK). Results using this sandwich ELISA Kit were determined according to the manufacturer’s instructions.

### Psychiatric outcomes

At each assessment time-point, participants completed the PANSS^57^, via a structured clinical interview designed to monitor symptoms of psychosis. The PANSS interview assesses positive and negative symptoms, and is widely considered the ‘gold-standard’ method of quantifying psychotic behaviour. Interviewers were trained in the completion of PANSS.

### Data analysis

Data analysis was performed using GraphPad Prism 8 software (version 8.0.1, 2018). At baseline, relationships between markers were determined using linear regression, and to assess any difference at baseline between the two groups, two-sample *t* tests were used. To assess changes between different time-points in the study, paired *t* tests were used. To compare relationships between marker pre-intervention/control period vs. mid or post, Pearson’s correlation coefficient was employed. Biomarkers were assayed in triplicate, with average values calculated. Outlying values were identified using the ROUT (Robust regression and Outlier removal) method (*Q* = 1%). Shapiro–Wilk test for normality was used for Gaussian distribution, with *α* significance level set at 0.05. Standardised mean difference (SMD) test was also used to assess effect size between time points and intervention groups, using the standard deviation of paired differences.

## Results

### Baseline

Twenty-two early intervention service users were recruited and randomised into the study, aged between 17 and 34 (average length of service use, at recruitment, was 19 months). Baseline characteristics are presented below (Table 1). From a potential caseload of 134, 67 patients did not meet eligibility criteria. Of the 67 remaining, with potential for inclusion into the study, 22 were randomised. Reasons for eligible patients not being randomised included discharge from the early intervention services, work/university time commitments, no interest in taking part in the study and an inability to make initial contact with the patient. Fifteen participants completed the trial, across the exercise (*n* = 7) and control (*n* = 8) groups. Peripheral biomarker analysis and symptom assessment was completed for all participants, whereas only four members of each intervention group were able to complete the MRS brain scan.

**Table 1:** Summary of baseline characteristics, for both exercise and control groups.

	Exercise		Control		
	Average	SD	Average	SD	*P* values
Weight (kg)	82.51	14.69	84.10	16.76	0.82
BMI (kg/m^2^)	25.45	3.71	25.49	4.65	0.98
Age (years)	23.45	3.75	26.10	5.74	0.22
Duration of illness (months)	20.56	9.99	18.00	11.70	0.61
Tobacco consumption	73%		64%		NA
Cannabis consumption	50%		91%		NA
Brain GSH (mM/kg) (tNAA)	0.254	0.061	0.275	0.044	0.75
Blood GSH (μM)	0.72	0.23	0.77	0.22	0.62
Comet (%)	3.20	1.52	2.54	1.41	0.31
TBARS (μM)	12.82	2.94	12.31	3.34	0.72
Protein carbonyl (nmol/mg protein)	138.44	42.69	150.52	136.05	0.79
IL-6 (pg/mL)	1.88	0.49	1.6	1.2	0.34
TNF-α (pg/mL)	83.69	13.99	87.25	12.14	0.54
CRP (ng/mL)	2.51	1.37	2.79	1.43	0.65
FRAP (μM)	294.64	57.01	270	36.68	0.26
BDNF (ng/mL)	52.53	20.91	40.56	26.87	0.64

*BDNF* brain-derived neurotrophic factor, *BMI* body mass index, *CRP* C-reactive protein, *FRAP* ferric-reducing ability of plasma, *GSH* glutathione, *IL-6* interleukin-6, *TBARS* thiobarbituric acid-reactive substances, *TNF-α* tumour necrosis factor-alpha.

### Brain and blood GSH

Peripheral whole-blood GSH increased by 6.13% in the exercise group, but significantly decreased by 24.37% in the control group (*p* = 0.04), in a group–time interaction (SMD = 0.47).

Ineligibility for scanning included foreign metal in the body (*n* = 2), anxiety/fear of enclosure in the scanner (*n* = 6) and injury preventing prolonged periods of time in the supine position (*n* = 1). Brain GSH concentration increased by 8.50%, and 8.80% in the exercise and control groups, respectively (SMD = −0.06). There was no significant change in brain GSH data as a result of 12 weeks of exercise (*p* = 0.55), but these data are limited by the small sample of the cohort that were able to be scanned (exercise group *n* = 4 and control group *n* = 4 for pre–post scans).

**Fig. 2:**
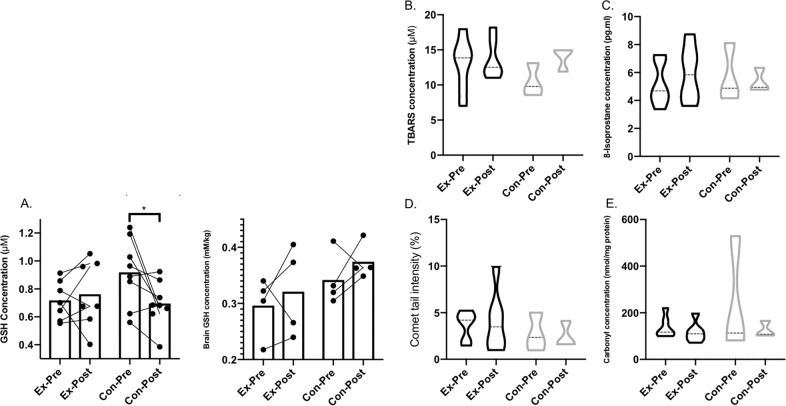
Changes in indices of antioxidant capacity and oxidative stress. **a** A comparison of whole-blood GSH concentration (μM) (left) vs. cerebral GSH concentration (mM/kg) (right), pre-intervention vs. post intervention. **b** Plasma thiobarbituric acid-reactive substances concentration (μM), a measure of lipid peroxidation. **c** Plasma 8-isoprostane concentration (pg/mL), a measure of lipid peroxidation. **d** Single-strand DNA damage from whole blood (%). **e** Plasma protein carbonyl concentration (nmol/mg protein). Both GSH measures are expressed as mean and standard deviation. All oxidative damage markers are expressed as median and interquartile range. * indicates a significant difference between pre- and post measures.

The Pearson’s correlation coefficient for the relationship between blood and brain GSH showed that in the control group, pre and post *r* values were 0.37 and 0.23, respectively, indicating weak positive correlation at both time points. The *r* value for the exercise group pre-intervention was 0.91, and post intervention 0.06. These changes indicate that, in this exercising group of participants, exercise affected peripheral and brain GSH regulation differently. Both blood and brain GSH changes are presented in Fig. 2. Additionally, in the exercise group, the relationship between GSH change and intensity minutes increase, as a result of being in the intervention, was strongly positively correlated, for both blood (*r* = 0.61) and brain (*r* = 0.68) measures.

### Markers of oxidative damage

Lipid peroxidation (assessed by TBARS (SMD = −0.67) and isoprostane concentration (SMD = 0.41)), protein oxidation (assessed by protein carbonylation (SMD = 0.31)) and DNA oxidation (assessed by strand breaks (SMD = 0.18)) are presented in Fig. 2. Exercise did not cause any significant changes in markers of oxidative stress. Repeated-measures analysis of variance (ANOVA) showed no difference between time points for each marker of oxidative damage. Due to the small sample size represented in this study, median values were used to better describe the central tendency of the data, since each measure had a wide range^58^.

### Inflammatory markers

The markers of inflammation CRP, IL-6 and TNF-α each changed differently in response to the exercise intervention. IL-6 concentration decreased between pre and post time-points in both groups: 25.98% in the exercise group and 43.30% in the control group (SMD = 0.25). CRP decreased by 10.29% in the exercise group and increased by 12.05% in the control group (SMD = −0.37). TNF-α decreased in both groups: 4.28% and 6.80% for the exercise and control groups, respectively (SMD = 0.13). Repeated-measured ANOVA showed no difference between time points for each inflammatory marker.

### BDNF concentration

Plasma BDNF content increased by 10.38% in the exercise group (*p* = 0.70), and declined by 17.52% in the non-exercising controls (*p* = 0.64). Despite the negligible increase in BDNF content in the exercise group over the full 12 weeks of the intervention period, between mid and post time-points in the exercise group, plasma BDNF concentration increased by a much greater margin of 41.58% (*p* = 0.12, SMD = 0.23).

### Cannabis and tobacco consumption

Subgroup analysis was undertaken in the exercise intervention group in participants who self-identified as regular cannabis smokers (*n* = 4), compared with non-smokers (*n* = 3). TBARS concentration decreased 1.30% in non-smokers, but increased 37.76% in smokers, and isoprostane concentration increased 5.22% in non-smokers compared to 17.72% in smokers. Protein carbonylation decreased 31.9% in non-smokers and decreased 5.84% in smokers. These effects may have been fortified by tobacco consumption, since all cannabis smokers were also tobacco smokers, and only one non-smoker consumed tobacco only. In an analysis of baseline values for tobacco smokers vs. non-smokers, there was no difference in any of the biomarkers assessed.

### Intensity minutes and markers of inflammation

**Fig. 3:**
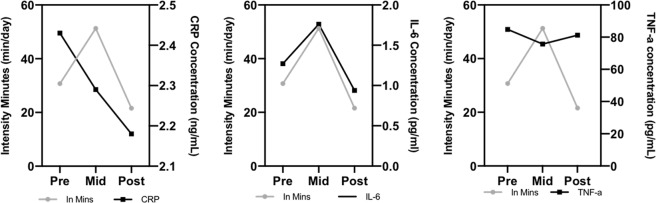
Garmin device measured ‘intensity minutes’, alongside markers of chronic inflammation CRP, IL-6 and TNF-α. The grey points represent ‘intensity minutes’, as derived from the Garmin devices, and the black points respective inflammatory cytokine concnetrations at pre-, mid- and post-intervention time-points.

The potential effect of quantity of exercise (intensity minutes) on markers of inflammation was assessed (Fig. 3). ‘Intensity minutes’, measured by the Garmin devices, are representative of the number of minutes of moderate-intensity physical activity that contribute to continuous bouts of activity lasting 10 min or more. CRP, IL-6 and TNF-α concentrations at pre-intervention, mid-point and post intervention were compared with intensity minutes per day. It can be seen in Fig. 3 that as intensity minutes increased from baseline to mid-intervention, resting IL-6 concentration also increased. Levels then fell as activity levels decreased post intervention. TNF-α plasma concentration decreased in response to increased activity, and increased once again post intervention. CRP decreased throughout the intervention period and was still decreased post intervention.

### Retention, exercise quality and psychopathology

Overall retention to the intervention was 68%, with attrition rates of 18% and 45% for the exercise and control groups, respectively. Four members of the exercise group dropped out, one immediately after baseline assessment, two during the first week of exercise and one after 10 weeks of intervention duration. Three members of the control group dropped out just before the 13-week post assessment was due. Attendance to the exercise sessions was 83% for the target two training sessions per week, and 41% for three sessions per week. The average duration of each exercise session was 37.74 min, and the mean number of sessions attended per week was 2.31, above the minimum target for the intervention. Exercise session data are summarised in Table 2. In the assessment of habitual activity at baseline, mid-intervention and post intervention, the exercise group demonstrated an increase from 30.7 min of activity per day to 51.3 during the intervention. This fell significantly to 21.6 min/day in the week following the intervention (*p* = 0.05). The control groups were similarly active at baseline, with an average of 25.6 min/day, which fell to 7.2 min/day.

**Table 2:** Summary of bout distribution and HR data for each separate activity type.

	Exercise bout modality							
	Swim	Run	Cycle	Tennis	Football	Squash	Badminton	Circuits
% Total sessions	18.3	15.3	3.5	14.9	16.8	4.5	5.9	20.8
Average HR (BPM)	117.5	136.3	137	125.5	139.4	124.5	124.1	121.3
95% CI	111.2–123.8)	(130.2–142.4)	117.5–156.5)	118.2–132.8)	(132–146.7)	(119.2–129.8)	(119.8–128.4)	(118.7–123.8)

*HR* heart rate, *BPM* beats per minute, *CI* confidence interval.

There were strong negative correlations between exercise intervention adherence and TBARS (*r* = −0.75, *p* = 0.08) and protein carbonyl concentration (*r* = −0.73, *p* = 0.09). The PANSS interview assessed symptomology across positive, negative and general psychopathology domains. In each of the three domains, exercise either conferred protection against declining scores observed in the control group or resulted in a much greater change between baseline and post-intervention measurement. There was a 1.44% increase in negative score in participants in the exercise group, and a 13.89% increase in participants in the control group (SMD = −0.33). There was a 17.31% decrease in positive symptoms of PANSS in participants in the exercise group, and a 7.83% decrease in the non-exercising controls (SMD = −0.18). Participants in the exercise group reported a 10.98% decrease in PANSS general score, compared to a 2.82% decrease observed in participants in the control group (SMD = −0.24). These data have been assessed alongside other functional measures that better shape the psychotic state, and the effects of the exercise intervention in Fisher et al.^45^.

## Discussion

This exploratory study demonstrated that 12 weeks of regular exercise training may be sufficient to elicit positive changes in biomarkers of redox status and inflammation in a cohort of young men with FEP. The study highlighted potential differences in the response of GSH to training in blood and brain. Although some results were not significant when analysing individual outcomes (due to small participant numbers), when taken together, the battery of outcomes suggest a shift in redox and inflammatory status. In many cases, exercise appeared to be protective of the negative changes that were observed in the control group, which are likely a result of disease status: either redox-linked disease progression, treatment or the impact the disease has on lifestyle factors. These are all representative indicators of poorer long-term outcomes for the patient.

### Brain GSH vs. peripheral GSH

The data presented suggest that blood and brain GSH responded differently to exercise. Exercise caused increased blood GSH, which is a common response^32^; however, no detectable change was seen in brain GSH in this study. There was no correlation between changes in blood and brain GSH, for either study group. It should be noted that the number of participants able to be scanned was less than those that were able to give a blood sample, and as such assessment of brain GSH was only possible in a subset of an already small sample of FEP patients. However, Rai et al.^59^ also reported differences in blood and brain GSH response to exercise. The BBB is impermeable to peripheral GSH and as such it is probable that the two pools of GSH are regulated separately, protecting the brain from peripheral chemical changes that could produce toxic effects to brain function and integrity^60^. The present study is the first to assess brain and blood GSH in response to exercise in a psychosis population.

In addition to the discordant regulation of GSH in brain and blood, there are a number of methodological considerations that are worthy of note. GSH determination by MRS does not discriminate between glial, neuronal or extracellular sources of GSH^61^, and since GSH concentration in astrocytes is 10 times that of neurons, differential neuronal regulation may be masked. The complexity of FEP, as with most mental illnesses, means that brain GSH has not been identified as having a causal role in disease pathogenesis, but rather perturbed GSH metabolism^26^ alongside other stress-related markers and antioxidants are believed to create the FEP phenotype. In the present study, there was most variation in brain GSH in the exercise group, and given that exercise training can cause changes in GSH, it may be appropriate to suggest that exercise may have indeed perturbed brain GSH, but participant number and confounding factors such as diet, smoking status and medication made it impossible to draw any conclusions from these data. From an exploratory standpoint, the data presented around GSH regulation warrants further investigation, given its critical role in health.

### Inflammation and redox status

No exercise intervention studies have yet assessed the effects of training on markers of inflammation in an FEP population. Exercise resulted in a positive change in many of the markers of redox status and inflammation. For example, CRP concentration reduced throughout the study in response to exercise (−10.29%) compared to an increase (12.05%) in the control group. This response is highlighted in Fig. 3, where exercise intensity minutes increased across the study, and CRP concentration fell. In a schizophrenia population, a 10-week intervention of high-intensity exercise resulted in a 66% decrease in plasma CRP^62^, matching the results shown in the present study. Other studies that have assessed CRP in psychotic disorders reported no change after exercise training^63,64^. IL-6 has been described as a myokine, which can act as a regulator of exercise-induced metabolic changes. IL-6 release is elevated during dynamic exercise^65^ and thus explains the observed increased plasma IL-6 concentration observed at mid-point during this intervention. A review paper by Gómez-Rubio et al.^66^ summarised that in schizophrenia, regular exercise leads to a reduction in disease-associated IL-6 elevation, as was observed at the post-intervention measure in the present study.

Exercise training conferred a protective effect for a number of blood biomarkers, including GSH, BDNF and TBARS. All of these measures demonstrated greater detrimental change in the control group, which may be associated with disease progression^67–69^. Increased TBARS, as a measure of lipid peroxidation, is thought to predate presentation of psychotic symptoms, in a comparison of unmedicated FEP patients, chronic schizophrenia patients and healthy controls^17^. In that study, the concentration of TBARS was greater in both patient groups, and greatest in the chronic schizophrenic population. This is in line with the results of the present study, which suggest that there is a disease course-dependent increase in lipid markers of free-radical-mediated damage, over time. BDNF, a regulator of neuronal health and synaptic plasticity, can be depleted in FEP, via an inflammatory-mediated pathway^70^. Increased circulating proinflammatory cytokine concentration, demonstrated in this clinical group, downregulates BDNF expression^71^. The current study observed a variety of responses to exercise in different inflammatory markers, which partially act to influence BDNF expression downstream. Most importantly, the protective effect of exercise was demonstrated as preventing the BDNF decline observed in the control group. BDNF concentration increased between the mid-intervention and post-intervention time-points, with a 22.03% decrease between baseline and mid-intervention, suggesting a lag-period between exercise initiation and the beginning of an adaptive response to training in this marker. Peripheral BDNF measures are used as a proxy for brain BDNF, since it is able to cross the highly restrictive BBB. Measurement in the plasma is representative of free BDNF that crosses the BBB, and a strong reflection of the influence of an intervention in the brain, rather than a serum measure that takes into account platelet secreted BDNF upon activation^72^. This study brings together many of the linked biochemical perturbations observed in FEP, and highlights the potential of exercise training to normalise indices of antioxidant depletion, oxidative damage, inflammation and neuronal function.

### Cannabis and tobacco interaction with redox markers

Regular use of cannabis in mental illness is far greater than the general population (6.6%)^73^, particularly in psychosis populations (23%)^74^. When data were assessed by subgroup in the current study, the positive effects of exercise that were seen in non-smokers were not observed in the subgroup of smokers. In a study assessing the redox effects of regular cannabis consumption in healthy young people, there was no difference in markers of lipid peroxidation or protein carbonylation between smokers and non-smokers^75^. However, there are many studies that show the negative effects of smoking on markers of redox status^76^. It is possible that changes in redox status as a result of cannabis smoking, followed by increases in markers of oxidative damage do contribute to the pathology of psychotic disease; however, more investigation of the specific effects of cannabis use on redox status in FEP is needed to draw any conclusions. Exercise did not confer complete protection for markers of free-radical medicated damage in the current FEP group. In addition, regular tobacco consumption was prevalent among the participants of this study (70% smokers, 30% non-smokers). Although the groups were too small to allow further analysis of the effects of tobacco smoking on adaptation to exercise, the protective effect of regular training on indices of oxidative stress in smokers has been highlighted. One 12-week intervention demonstrated significant improvements in total antioxidant capacity, GSH peroxidase and GSH reductase enzymes, alongside a reduction in lipid peroxidation products in smokers only, with no adaptive change in a non-smoking control group^77^. Although baseline oxidative stress is normally higher in smokers^78,79^, the benefits of an exercise intervention are particularly salient in this FEP population, given the high percentage of the population who habitually smoke tobacco.

### The impact of exercise on symptoms of psychosis

The changes in biomarker described herein were accompanied by a positive change in symptom profile as a result of exercise. The observed changes in symptoms of FEP indicate that the current intervention design was sufficient to elicit beneficial changes in functional status (PANSS symptomology), in addition to changes in biomarkers of redox status and inflammation as a result of exercise training. This is in concordance with the current literature; exercise has been shown to be superior to control conditions in improving indices of symptomology, quality of life and depressive symptoms^27^. Additionally, another meta-analysis showed the value of 90 min of moderate-intensity exercise per week (below the World Health Organisation recommendation of 150 min moderate–vigorous-intensity exercise per week) in improving psychiatric symptoms^80^.

### Limitations

The principle limitation in this study was a small sample size, rendering many of our time-point and inter-marker comparisons statistically insignificant. However, the current study did observe a measurable change in redox status and clinical outcome measures. Not all participants were able to be assessed for brain GSH do to ineligibility or inability to enter the brain scanner. The main barrier to scanning was anxiety of participants to be scanned, but there were some incidences of metal compounds in the body, or injury preventing prolonged time in the supine position. Lastly, despite the opportunity to undertake a variety of indoor-based activities, poor and unpredictable weather in the United Kingdom was a factor in participants’ willingness to engaging in the intervention. This effect was particularly salient during the winter months. Confounding factors such as adherence to the intervention, body mass index and cannabis consumption may have affected the outcomes of this exercise intervention, together with potential contributions from antipsychotic medication and duration of illness.

## Conclusion

This study, in male patients with FEP, highlighted the beneficial effects of exercise training on markers of redox homeostasis. The study showed that 12 weeks of moderate-intensity exercise training was sufficient to reduce markers of lipid oxidation and increase antioxidant status. In many of the markers assessed in this study, exercise was able to arrest the changes that were seen in the control group, which would usually be associated with FEP. Future studies should use these data to explore more definitive outcomes, given the feasibility of such an intervention, and the observed change in markers that describe several aspects of disease pathology.
